# Ethical considerations when co-analyzing ancient DNA and data from private genetic databases

**DOI:** 10.1016/j.ajhg.2023.06.011

**Published:** 2023-08-03

**Authors:** Éadaoin Harney, Kendra Sirak, Jakob Sedig, Steven Micheletti, Roslyn Curry, Samantha Ancona Esselmann, David Reich

**Affiliations:** 1 23andMe, Inc.; Sunnyvale, CA 94043, USA; 2 Department of Human Evolutionary Biology, Harvard University; Cambridge, MA, 02138, USA; 3 Department of Genetics, Harvard Medical School; Boston, MA, 02115, USA; 4 Howard Hughes Medical Institute, Harvard Medical School; Boston, MA, 02115, USA; 5 Broad Institute of MIT and Harvard; Cambridge, MA, 02142, USA

## Abstract

Ancient DNA studies have begun to explore the possibility of identifying identical DNA segments shared between historical and living people. This research requires access to large genetic datasets to maximize the likelihood of identifying previously unknown, close genetic connections. Direct-to-consumer genetic testing companies, such as 23andMe, Inc., manage by far the largest and most diverse genetic databases that can be used for this purpose. It is therefore important to think carefully about guidelines for carrying out collaborations between researchers and such companies. Such collaborations require careful consideration of ethical issues, including policies for sharing ancient DNA datasets, and ensuring reproducibility of research findings when access to privately controlled genetic datasets is limited. At the same time, they introduce unique possibilities for returning results to the research participants whose data are analyzed, including those who are identified as close genetic relatives of historical individuals, thereby enabling ancient DNA research to contribute to the restoration of information about ancestral connections that were lost over time, which can be particularly meaningful for families and groups where such history has not been well documented. We explore these issues by describing our experience designing and carrying out a study searching for genetic connections between 18^th^−19^th^ century enslaved and free African Americans who labored at Catoctin Furnace, Maryland, and 23andMe research participants. We share our experience in the hope of helping future researchers navigate similar ethical considerations, recognizing that our perspective is part of a larger conversation about best ethical practices.

## eTOC

The search for genetic connections between ancient and living individuals benefits from access to large genetic databases, like those maintained by genetic testing companies. We discuss the ethical issues we considered during our recent study of African Americans from Catoctin Furnace Maryland, who were compared to 23andMe, Inc’s genetic database.

We recently identified genetic relationships between 18–19^th^ century African Americans from Catoctin Furnace Maryland, and over forty thousand consenting research participants in 23andMe, Inc.’s genetic database ^1^. These connections include distant relatives and possible direct descendants of the Catoctin individuals. This represents one of the first studies to our knowledge where identical-by-descent (IBD) segments of the genome (segments of DNA that are shared by two or more people because they have been inherited from a recent common ancestor) have been identified between historical individuals and their previously unknown living relatives by leveraging a dataset held by a private company ^2^. In breaking this new ground, this study encountered ethical issues that had not been addressed in the existing literature on ethics in ancient DNA (aDNA) ^3–10^, although related concerns surrounding the rise of direct-to-consumer genetic ancestry testing have been discussed broadly in the social sciences literature ^11–14^. Here we discuss the approach we took in response to these issues with the hope that our experiences help inform future work by researchers in the field of ancient DNA, genetic ancestry companies interested in contributing to research collaborations or creating product features involving ancient DNA data, and anthropologists and curators who serve as stewards of human remains.

The genetic analyses of the remains that are reported in Harney et al. were carried out at the request of the Catoctin Furnace Historical Society (CFHS) (led by current president, Elizabeth Comer), which advocated for further study of the Catoctin remains in partnership with the African American Resources and Cultural Heritage Society (AARCH) of Frederick, Maryland, later joined by individuals identified by researchers at CFHS as descendants of two African Americans who labored at Catoctin Furnace. The remains of the Catoctin individuals have been under the stewardship of the Smithsonian Institution since 1980, following their excavation from the African American Cemetery at Catoctin Furnace in advance of planned highway construction. Preliminary anthropological analyses of the historical Catoctin individuals were performed following their excavation to determine the identity of those buried in the undocumented cemetery ^15,16^, but these analyses were limited in scope. They failed to meet the key goals of the CFHS and AARCH, which were to “identify a descendant community for the Catoctin African-American workers (enslaved and free); to connect the individuals within the cemetery to their ancestral roots in Africa; and to share the discovery process and its results with the public” ^17^.

In 2016, CFHS received a Maryland Heritage Areas Authority grant to undertake a forensic re-analysis of the Catoctin individuals ^18^, initiating a new investigation into Catoctin using a variety of anthropological and biomolecular tools. This work has contributed to community and national level conversations about the role that scientific approaches can play in restoring information about the lives of enslaved people that would otherwise be lost to history ^19,20^. In its first phase, researchers at the Smithsonian Institution partnered with the CFHS to study the demography and life history of the Catoctin individuals using osteological and isotopic approaches ^21^.

In the next phase of the project, this research team sought to investigate the ancestral origins, familial relationships, and legacy of the Catoctin individuals through genetic analysis. Members of the Reich Laboratory at Harvard University were invited to join the study team based on their expertise in aDNA recovery and population genetic analyses. Sampling for aDNA was carried out with the approval of the Anthropology Collections Advisory Committee (CAC) at the Smithsonian Institution, and the sequenced ancient DNA were reported in a technical note ^22^ and made fully publicly available following open science best practices ^23^ and the Smithsonian Institution’s curatorial requirements ^6^ However, without access to a diverse genetic database, it would have been impossible to effectively search for a Catoctin descendant community using genetic approaches. Access to a database that was larger and more diverse than any that is publicly available provided an extremely powerful and otherwise unattainable tool ^13^ for addressing the request of the CFHS to search for a descendant community because the number of connections that can be found between past and present-day people is directly proportional to the number of people in the database and also is dependent on the diversity of people in that database. The research team identified 23andMe as an appropriate partner due to the size of their database, which contains genome-wide data from ~12.8 million people (more than an order of magnitude more individuals than any publicly available dataset) ^24^, the high diversity of people represented in the 23andMe dataset, 23andMe’s experience developing population genetic tools to identify genetic connections through identity by descent analysis ^25–27^, and 23andMe’s track-record of publishing open-access research by leveraging its database ^28,29^. Critically, over 80% of 23andMe customers have provided consent for their de-identified survey answers and genetic data to be used for research purposes and can be recontacted based on research findings for a variety of purposes, including to answer follow up questions or to be invited to participate in additional, in-depth studies ^12,30^. This also means that, in theory, it could be possible to return results to descendants identified over the course of the study (although how best to do this if at all is still under consideration, and no recontacting based on the results of Harney et al. has yet occurred). Therefore, with support from CFHS, AARCH, a partnership was explored between 23andMe and the research team, in which ethics remained at the forefront of discussions.

We addressed these ethical considerations in a robust way that respects core academic principles through a formal written agreement between researchers at Harvard University and the Howard Hughes Medical Institute (HHMI) who were responsible for generating and sharing the Catoctin dataset, and 23andMe, who were responsible for comparing the Catoctin data with the 23andMe database. During the course of the study design and contract negotiations, we identified three key areas that warranted special consideration: data sharing, study reproducibility, and return of results.

## Sharing of ancient DNA datasets

To date, the vast majority of aDNA data have been made fully publicly available in accordance with the principles of open science ^23,31^. However, for datasets that are closely associated with living descendants and other stakeholder communities, some researchers have advocated for restricting access ^3,4,32,33^. Restrictions on data access that have been applied have included that they can only be used for non-commercial purposes, or in some cases, only for replication purposes, and that explicit permission from community stakeholders may be required before sharing data for additional research purposes ^e.g. 34–36^.

While the best data sharing policies for aDNA data may be context specific, all authors agree that a scenario in which a for-profit company–or any research or commercial entity that may benefit financially, either directly (e.g., by charging fees to access research results) or indirectly (e.g., career advancement), by restricting access to data–retains exclusive, unrestricted access to an aDNA dataset following study completion would be inappropriate. Instead, clear access guidelines should be created that apply to all parties who would like to access the data. In some for-profit companies, like 23andMe, there is a distinction between that organization’s research and their commercial activity. Thus, even if data are available for non-commercial purposes only, this need not prevent research-focused collaborations with for-profit organizations. Regardless of the data sharing policy that is implemented, it is critical to ensure that the details and implications of this policy are thoroughly communicated to all stakeholders involved in the study, as was done as a part of Harney et al.

The collaboration agreement between Harvard, HHMI and 23andMe ensures that any non-public aDNA dataset shared with 23andMe can only be used for research and publication purposes. The data can only be used by 23andMe for other purposes after it is made fully publicly available. In the case of the Catoctin project, the terms of the sampling agreement set forth by Smithsonian Institution required the Catoctin aDNA dataset to be made fully publicly available upon publication, or within three years of sampling. To meet the terms of this agreement we released a technical report describing the Catoctin data in June 2022 ^22^, over a year prior to publication of the joint analyses of the historical genomes and 23andMe research participant data in Harney et al.

## Challenges in ensuring reproducibility in studies involving private genetic databases

The 23andMe genetic database, like many other private genetic databases, is subject to restrictions on individual-level data sharing that make publishing fully reproducible studies challenging ^30^. Some areas of population genetics, particularly genome-wide association studies (GWAS), have developed standards for sharing summary statistics that do not contain any individual level information, thereby respecting the privacy restrictions required of these private genetic databases while also enabling some degree of reproducibility ^e.g. 37,38^. However, for IBD-based studies that require direct comparisons between individual genomes, there is no corresponding approach that would enable a similar level of reproducibility of study results through the release of summary statistics. There is no perfect solution for this problem, but weighed against this is the fact that leveraging a genetic database as large as 23andMe’s in aDNA powers research that otherwise would be impossible. In fact, it is possible that 23andMe’s high research participation rate can be attributed in part to the strong privacy protections that are offered to research participants, including the ability to withdraw consent at any time and restrictions on individual-level data sharing without additional explicit consent.

Through a series of discussions between researchers and legal advisors at Harvard, HHMI, 23andMe, and later editors at the journal where Harney et al. was published, we developed a two-phase approach to maximize the ability of researchers to independently replicate the results of our study.

1. We included genetic data drawn from several publicly available datasets, including the 1000 Genomes Project ^39^ and 23andMe’s African American Imputation Panel ^40^, in our study dataset. Whenever possible, we report the results of each analysis for these subsets of individuals. This approach makes it possible for researchers who wish to investigate the accuracy of the results to reproduce fully a portion of the study using these publicly available datasets.
2. For all analyses that cannot be fully reproduced using the above method, 23andMe committed to rerun comparisons upon request by academic and non-profit researchers on reasonable terms for a period of up to seven years from the date of publication or for as long as the study co-authors are employed at 23andMe, whichever is shorter. This commitment is time-limited, in recognition that changes to data storage formats and technology (and the roles held by researchers) over time mean that no studies are likely to be fully reproducible indefinitely. This is similar to the common academic institutional requirement that laboratory notebooks be retained to support reproducible research for a number of years, but not indefinitely ^e.g. 41^.

## Return of results to community stakeholders and research participants

In recognition of the importance of involving community stakeholders in the research process, the formal collaboration agreement enabled sharing of preliminary 23andMe research results not only with the external research partners at Harvard and HHMI who signed the agreement, but also with other research partners, including those at the Smithsonian, and members of relevant stakeholder communities, including CFHS, AARCH and previously identified descendants of the Catoctin individuals.

We also recognize that 23andMe’s re-contactable research cohort presents an opportunity to explore options to return results not only to stakeholder communities with known ties to the Catoctin individuals, but also to the research participants whose genetic information was used in the study – an option that is not typically available for studies performed using publicly available datasets. Although the research consent process makes it clear to 23andMe research participants that they should not expect to have individual-level results returned to them as part of the research process, it leaves open the possibility that research could aid in the development of future 23andMe features through which results could be returned ^30^.

### Ethical considerations for future feature development by genetic ancestry companies

Although the aims of Harney et al. were purely research focused, over the course of the study we considered how a feature or report in the 23andMe user interface could be used to help realize one of the original goals of CFHS and AARCH: to identify and foster a Catoctin descendant community. In what follows, we discuss some issues that we believe would be critical for genetic ancestry companies to address when designing a product feature that reports IBD connections shared between customers and ancient or historical individuals.

#### Providing opportunities to opt-in to receive results

Although use of re-contactable research cohorts in studies like Harney et al. provide a unique opportunity to return results to research participants, participants may not wish to learn about these connections for a variety of reasons. For instance, in the case of Catoctin, learning about genetic connections to a direct ancestor or distant relative who was enslaved could be a painful experience that evokes known family trauma or reveals an unknown personal connection to slavery in the United States. In the case of Harney et al., while community stakeholders expressed an interest in learning about any potential genetic connections they might share with the Catoctin individuals, they were also clear that they would like to be given the opportunity to choose to receive this information rather than having it shared with them without warning. When returning results to research participants or other customers in their database, genetic ancestry companies should give customers the opportunity to actively opt in to receive this information and provide adequate information to help customers anticipate what they might learn by doing so. In making this recommendation, we acknowledge that users who opt not to receive this information from genetic ancestry companies directly may still learn about their likely genetic connections to historical individuals via relatives who do choose to opt in or explore their connections using other means. Our recommendation to include an opt-in step before returning results to customers mirrors the consent that customers at 23andMe and other genetic ancestry companies give before receiving information about living genetic relatives ^42^.

#### Avoiding biological gatekeeping to community membership

While identifying a Catoctin descendant community using a genetic approach was one of the goals of Harney et al, genetic connections are only one of a variety of ways through which stakeholders can be connected to historical communities, and may not be the most important form of connection. Other forms of connection include non-genetic familial relationships, through shared culture or heritage, or through a collective kinship bond that does not depend on recent shared ancestry. For instance, key community stakeholders associated with the Catoctin individuals self-identify as members of a “collective” descendant community that was intentionally created by the CFHS and AARCH to honor and protect the Catoctin individuals ^1^. Although this collective descent community was created because there was no other known community that claimed to have a connection to the Catoctin individuals, any newly identified descendant community (connected via genetics, genealogy, or any other means) would not diminish the importance of this established community.

When returning results to research participants who share a genetic connection to a historical population, it could be tempting for genetic ancestry companies to emphasize these genetic connections over the many other ways through which stakeholders can be connected to a historical group. However, when returning results to research participants or other customers, genetic ancestry companies should be explicit that genetic connections are not necessarily more important than other types of connections. Research on genetic connections to historical individuals has the potential to contribute to the biologization of identity whether intentionally or not. Thus, when returning results, genetic ancestry companies should take care to identify and describe the presence of existing stakeholder communities. In the case of Catoctin, existing stakeholders have been explicit that they are eager to engage with genetic relatives of the Catoctin individuals. In other cases, genetic relatives who lack any other connection to a historical group may not be welcomed by the existing stakeholder community on the basis of their genetic results alone.

#### Prioritizing accuracy when returning results

When designing product features that reveal genetic connections to historical people, it is critical to use approaches that are optimized to work on aDNA data. Although many population genetic tools are capable of generating output when applied to aDNA data, it should not be assumed that these results are meaningful unless the performance of those tools on aDNA data has been rigorously assessed—paying particular attention to the impact of missing data, C-to-T misincorporations, low confidence genotype calls, and, when applicable, imputation errors ^43^. Approaches that do not account for the unique characteristics of aDNA data have the potential to be highly inaccurate, thereby failing to identify true genetic connections (“false negatives”) and incorrectly identifying genetic connections in cases where none exist (“false positives”). It is therefore critical to identify optimal approaches for analyzing aDNA data and minimum quality standards for the type of aDNA data that can be analyzed. For instance, in the Catoctin study, we optimized an approach for identifying IBD connections that can be confidently applied to aDNA data with over 1x coverage. We recommended caution when interpreting the results of data with less than 1x coverage and did not report results for data with less than 0.5x coverage.

In many cases the identification of a genetic connection to a historical individual may impact customers in profound ways—particularly in cases where customers may have sought out genetic ancestry testing in order to fill gaps in their family and community history. For instance, many descendants of enslaved Africans in the United States have turned to genetic ancestry testing to learn about their family history ^11,44–46^. Discovering a shared genetic connection to a historical African American from Catoctin or another site could therefore be extremely impactful, while returning inaccurate or misleading results has the potential to cause confusion and other harms ^14,46^. We therefore believe that methods for comparing aDNA to customers should be rigorous to avoid false positives and if an analysis identifies only weak affiliations–for example, due to sharing a common ancestor hundreds or thousands of years ago–genetic ancestry companies should make this clear and not overstate the likelihood (or in the case of more ancient individuals, the uniqueness) of direct genealogical connections. A methodological tradeoff associated with reducing the false positive rate for any well-optimized analysis is an increase in the frequency of false negatives, therefore genetic ancestry companies should also be sure to communicate that the lack of IBD sharing cannot be interpreted as conclusive evidence that there is no shared genetic relationship.

#### Taking responsibility for returning results in a comprehensible way

Genetic ancestry companies should also recognize that returning inaccurate results or returning results in a confusing way may actively harm community stakeholders, people or organizations who serve as stewards for the human remains, those who serve in other roles that involve educating the public about specific historical individuals or sites, or broader public understanding. In the case of Catoctin, while groups like CFHS, AARCH and researchers at the Smithsonian Institution are committed to helping potential descendants and other stakeholders learn about Catoctin, they should not have an added responsibility of helping customers of genetic ancestry companies interpret their genetic results. Reports generated by genetic ancestry companies should be created with enough educational content and detail so that customers with shared genetic connections can interpret their results without the need for guidance from outside organizations. In fact, genetic ancestry companies may wish to consult with members of these organizations to ensure that any product features that involve these historical populations are represented accurately and clearly. But in doing so, they should again be careful not to place the burden of responsibility for providing comprehensible results on these organizations.

## Conclusion

We believe that joint analysis of historical genomes and data in genetic databases controlled by for-profit genetic ancestry companies represents an exciting new direction for aDNA research. As ancient DNA research continues to leverage the power of ever-growing datasets, all parties involved in this work must make a concerted effort to predict ethical issues that may arise and proactively design an ethically-sound strategy to ensure that sensitive genetic data remains protected. The continued application of ancient DNA technology to new contexts is inevitable, and we are encouraged by the robust conversation about best ethical practices that provided a foundation for this work^3–14^. The present paper aims to contribute to this body of literature by highlighting the specific issues we identified as ethical challenges in carrying out Harney et al. in the hope that it might help future researchers navigate similar ethical considerations in projects like this one going forward. Future work will certainly add to and amend the guidance we provide, which is part of moving forward the ethics of the rapidly growing field of ancient DNA research.
